# A comparative evaluation of icon® resin infiltrant and embrace^TM^ wet bond as pits and fissure sealant: A one year follow up study

**DOI:** 10.6026/973206300212101

**Published:** 2025-07-31

**Authors:** Deepthi Priyanka G.N, Suzan Sahana, Niranjani Krothapalli, Ramakrishna Juvva, Snigdha Gavini, Neelima Cherukumalli Kapalavayi

**Affiliations:** 1 Department of Pedodontics and Preventive Dentistry, Private Practitioner, Kadapa, India; 2 Department of Pedodontics and Preventive Dentistry, St Joseph Dental College, Eluru, India; 3 Dental Director, Jefferson Dental & Orthodontics, Texas, USA, 77373; 4 Department of Pedodontics and Preventive Dentistry, St Joseph Dental College, Eluru, India; 5 Department of Conservative Dentistry and Endodontics, St. Joseph Dental College, Eluru, India; 6 Managing Dentist at Brident Dental and Orthodontics; Texas, USA

**Keywords:** Pits and fissure sealant, resin infiltrant, caries prevention, self-priming resin

## Abstract

Occlusal surface of posterior teeth are highly susceptible to caries due of their anatomy. Therefore, it is of interest to evaluate and compare the retention and caries prevention ability of resin infiltrant ICON® with the moisture tolerant self - priming resin Embrace^TM^ Wet Bond. A non- significant difference was observed (p > 0.05) for both materials with respect to retention after 1 month, 3 months, 6 months and 9 months intervals. A significant difference was found (p<0.03) between ICON® resin infiltrant vs Embrace^TM^ Wet Bond with respect to the retention after 12 months.

## Background:

Occlusal surfaces with deep pits and fissures are an ideal site for the retention of food remnants. The complex morphology of the occlusal surface limits the mechanical plaque removal and proven preventive measures thus need for specific prevention of occlusal surfaces risen [1]. So, maintenance of oral hygiene, fluoride therapy, use of pit and fissure sealant seems to be the best measures in preserving the of the tooth structure from caries. Pit and fissure sealants play an important role in caries preventive strategies [2]. Currently, there are 2 basic types of sealants available *i.e.* Resin and glass ionomer sealants. Resin based materials are most commonly used due to their high retention rates [3]. Placement of resin based sealant is very technique sensitive and is influenced by factors such as presence of moisture, patient cooperation, operator variability and contamination of the operating field. However, the biggest drawback of resins sealant is its extreme sensitivity to moisture, as they are Bis GMA based materials that are primarily hydrophobic in nature [4]. Therefore, it is of interest to evaluate and compare the retention and caries prevention ability of resin infiltrant ICON® with the moisture tolerant self - priming resin Embrace^TM^ Wet Bond.

## Materials and Methods:

The present study was conducted on a group of children who attended the outpatient department of Pedodontics and Preventive Dentistry, St. Joseph Dental College and Hospital, Eluru, Andra Pradesh, India. The Guardian/Parents of the participants signed individual informed consent forms. The study was approved by the Institutional Ethical Committee.

## Procedure:

A randomized, single blinded clinical controlled trial was designed to assess the clinical performance of self-priming hydrophilic resin Embrace^TM^ wet bond and ICON® resin infiltrant as pit and fissure sealants. A total of 100 children aged between 6-8 years having deep pit and fissures on mandibular first permanent molars bilaterally were selected for the study. Individual caries risk was calculated by dmft index for each patient. Children with dmft score 2-5 were included in the study. A split mouth design was used in this study. The mandibular first permanent molars were cleaned using fluoridated pumice followed by isolation with rubber dam. ICON® resin infiltrant was placed to seal the pits and fissures on teeth of one side of the mouth and Embrace^TM^ wet bond sealant was placed on the other side following the manufacturer's instructions. The participants were evaluated at 1, 3, 6, 9 and 12 months after application of sealant to check for the retention of the material and also the occlusal caries incidence, by two calibrated independent evaluators. Sealants were categorized as completely retained, partially retained, or completely lost. The retention rate was assessed based on the criteria proposed by Tonn and Ryge 1982 and caries incidence by CCC Sealant evaluation system. All the remaining carious lesions if any were restored in the subsequent visit. The teeth were assessed for retention objectively by making an elastomeric impression. The prepared casts were evaluated and compared from baseline *i.e.* first visit to final recall visit.

## Scores for evaluation are as follows:

[1] Sealant present on all pit & fissure system of the tooth - (Score 0)

[2] Partially Retained - (Score 1)

[3] No sealant present - (Score 2)

## Statistical analysis:

All the data were tabulated and subjected to statistical analysis. A non-parametric sign test, unpaired t test and one way ANOVA test with SPSS version 19 software were used for analysis.

## Results and Discussion:

## ICON® resin infiltrant group:

Among the 100 teeth treated with ICON® resin infiltrant 88 teeth at 1 and 3 months, 84 teeth at 6 months, 50 teeth at 9 months and 16 teeth at 12 months showed complete retention (CR). 11 teeth at 1 and 3 months, 16 teeth at 6 months, 48 teeth at 9 months and 56 teeth at 12 months showed partial loss (PL). Complete loss of material observed from 9 months after application.

## Embrace^TM^ Wet Bond group:

In 100 teeth treated with Embrace^TM^ Wet Bond 84 teeth at 1 and 3 months, 76 teeth at 6 months, 40 teeth at 9 months and 8 teeth at 12 months showed complete retention (CR). 12 teeth at 1 and 3 months, 24 teeth at 6 months, 36 teeth at 9 months and 28 teeth at 12 months showed partial loss (PL). Complete loss of material observed from 9 months after application. The above results showed that there is non-significant difference (p > 0.05) between both the material with respect to retention when compared at 1, 3, 6 and 9 months intervals. On the contrary; at 12 months only 28 teeth out of 100 showed complete loss (CL) in ICON® resin infiltrant group whereas in Embrace wet bod group 64 teeth showed complete loss (CL). So at 12 months; significant difference (p<0.03) was found between ICON® resin infiltrant vs Embrace^TM^ Wet Bond with respect to the retention. The occlusal surface of posterior teeth are highly susceptible to caries because of the pits and fissures anatomy [5]. The utilization of an occlusal barrier which isolates the occlusal surface from the surrounding environment and thus limits the onset of caries resulted in the emergence of the sealant systems [6]. Fissure sealing has been shown to be an effective evidence-based caries preventive [7]. Non-sealed teeth need to be restored approximately 50% more frequently compared to the teeth which are sealed. Sealants are effective caries preventive agents as long as they remain bonded to teeth [8]. The different methods which improve sealant retention are: cleaning of the occlusal surface with hydrogen peroxide prior to sealant application, pumice prophylaxis, air polishing, mechanical preparation of fissures and air abrasion [9, 10- 11]. However, in spite of the proven efficacy and relative ease of application of sealant materials, retention is the main determinant in maintaining a sealant's caries-preventive effect [10]. The retention of sealants depends upon the ability of the resin sealant to completely fill the pits and fissures and or morphological defects on the occlusal surface and remain completely intact and bonded to enamel surfaces [12]. Subramaniam *et al.* [13] reported 4.5% complete loss of Embrace^TM^ Wet Bond at the end of 3 months which was greater than the value observed in the present study (0%). In the present study both the sealant materials showed 12% loss at 1st and 3rd month follow up and by the end of 6 months the loss observed for ICON® resin infiltrant was 16% whereas 24% for Embrace^TM^ Wet Bond which was in accordance with Yengopal *et al.* [14]. Comparing the complete loss of sealant between 6 months to 1 year both groups showed a similar pattern of retention with gradual decrease from 84% & 76 % to 16% and 8% respectively. Change in partial loss of the material for ICON® resin infiltrant and Embrace^TM^ Wet Bond were 12% to 56% and 12% to 28%. The results of Embrace^TM^ Wet Bond are in accordance with the findings of Schlueter *et al.* 2013; they obtained 60% of partial loss and 13% complete loss of Embrace^TM^ Wet Bond in 1 year [9]. In the present study, Embrace^TM^ Wet Bond showed 8% complete retention and 56% partial retention and 28% complete loss which were much higher than the earlier reports [15, 16-17]. Higher retention of ICON® resin infiltrant when compared to Embrace^TM^ Wet Bond in the present study can be correlated with Washburn equation [18]. Pit and fissure sealants are the major cornerstone of preventive dentistry. In the present study the ICON® resin infiltrant exhibited better retention than Embrace^TM^ Wet Bond. Further research to explore the effect of ICON® resin infiltrant with larger sample size and follow up for longer periods are necessary to confirm its practical applicability in paediatric dentistry.

## Financial support and sponsorship:

Nil.

## Conclusion:

We show that retention of ICON® resin infiltrant on the occlusal surfaces is more effective when compared to conventional pit and fissure sealant. Apart from its indication for masking of White Spot Lesions it can serve as an effective modality for the prevention of dental caries on the pit and fissures of first permanent molars.

## References

[1] Gugnani N (2012). Contemp Clin Dent..

[2] Ahovuo-Saloranta A (2013). Cochrane Database Syst Rev..

[3] Simonsen R.J (2002). Pediatr Dent..

[4] Welbury R (2004). Eur J Paediatr Dent..

[5] Bhatia M.R (2012). J Indian Soc Pedod Prev Dent..

[6] Iyer R.R (2013). Chin J Dent Res..

[7] Feigal R.J (2002). Pediatr Dent..

[8] Paris S (2010). J Dent Res..

[9] Schlueter N (2013). Clin Oral Investig..

[10] Paris S (2014). J Dent..

[11] Agrawal A, Shigli A (2012). J Indian Soc Pedod Prev Dent..

[12] Ninawe N (2012). Contemp Clin Dent..

[13] Subramaniam P (2008). J Indian Soc Pedod Prev Dent..

[14] Yengopal V (2009). J Oral Sci..

[15] Luca-Fraga L.R, Pimenta L.A (2001). Quintessence Int..

[16] Wendt L.K (2001). Community Dent Oral Epidemiol..

[17] Oba A.A (2012). Med Princ Pract..

[18] Cannon M.L, Comisi J.C (2013). Compend Contin Educ Dent..

